# Video stimuli reduce object-directed imitation accuracy: a novel two-person motion-tracking approach

**DOI:** 10.3389/fpsyg.2015.00644

**Published:** 2015-05-19

**Authors:** Arran T. Reader, Nicholas P. Holmes

**Affiliations:** ^1^School of Psychology and Clinical Language Sciences, University of ReadingReading, UK; ^2^School of Psychology, University of NottinghamNottingham, UK

**Keywords:** imitation, two-person, kinematics, grip aperture, joint angles, ecological methods

## Abstract

Imitation is an important form of social behavior, and research has aimed to discover and explain the neural and kinematic aspects of imitation. However, much of this research has featured single participants imitating in response to pre-recorded video stimuli. This is in spite of findings that show reduced neural activation to video vs. real life movement stimuli, particularly in the motor cortex. We investigated the degree to which video stimuli may affect the imitation process using a novel motion tracking paradigm with high spatial and temporal resolution. We recorded 14 positions on the hands, arms, and heads of two individuals in an imitation experiment. One individual freely moved within given parameters (moving balls across a series of pegs) and a second participant imitated. This task was performed with either simple (one ball) or complex (three balls) movement difficulty, and either face-to-face or via a live video projection. After an exploratory analysis, three dependent variables were chosen for examination: 3D grip position, joint angles in the arm, and grip aperture. A cross-correlation and multivariate analysis revealed that object-directed imitation task accuracy (as represented by grip position) was reduced in video compared to face-to-face feedback, and in complex compared to simple difficulty. This was most prevalent in the left-right and forward-back motions, relevant to the imitator sitting face-to-face with the actor or with a live projected video of the same actor. The results suggest that for tasks which require object-directed imitation, video stimuli may not be an ecologically valid way to present task materials. However, no similar effects were found in the joint angle and grip aperture variables, suggesting that there are limits to the influence of video stimuli on imitation. The implications of these results are discussed with regards to previous findings, and with suggestions for future experimentation.

## Introduction

To effectively imitate, visual information about an action must be combined or compared with a representation of the movements necessary to complete the action (Molenberghs et al., 2009). In relation to this, imitation research has often gone hand-in-hand with studies relating to the proposed human “mirror neuron system” (MNS). The MNS provides a potential basis for the ability to combine visual information with an internal representation of the observed movement. Early research using single cell recording found that neurons in the macaque premotor cortex activated both during the performance of an action, and when the same action was observed in another individual (di Pellegrino et al., 1992; Gallese et al., 1996), hence the term “mirror neurons.” Observed actions are often related to those encoded in one's own motor repertoire (Oztop et al., 2013), which may in turn provide an insight into the aims of the action and the potential intentions of the observed individual. Much research has aimed to establish the existence of a human MNS—a frontoparietal network that activates during both action observation and performance (Iacoboni et al., 1999), supported by neuroimaging (Molenberghs et al., 2009) and neurophysiological (Naish et al., 2014) evidence. The MNS likely plays a vital role in imitation, and it is possible that imitation relies in part on accurate, unconstrained observation of another's actions. It follows that any methodology impeding the natural observation of actions is likely to result in less effective understanding of the action, and therefore less effective imitation.

Surprisingly, little research addresses the reliability of video stimuli for experiments on the MNS, social interaction, or imitation. Limb movement is complex and three dimensional, and its observation could be undermined by a 2D viewing set-up (i.e., as observed on flat computer monitors or projection screens). This is particularly worth consideration when much of the research into imitation has used video stimuli presented to a group of solitary observers. There are discrete differences between direct observation of a scene, and observing the same scene reconstructed on a 2D surface (e.g., a computer monitor or projected image). For example, information from binocular disparity in a 3D scene is lost when presented in 2D. The treatment of 2D and 3D stimuli by the visual system varies wildly (Patterson, 2009). Additionally, there is little understanding of how the motor system responds to video vs. real life scenes.

Järveläinen et al. (2001) suggested that video feedback may not be the most appropriate medium for studying social interaction, particularly in an object-directed context. They focused on one proposed element of the human MNS—the primary motor cortex (Hari et al., 1998). Using magnetoencephalography (MEG) they recorded magnetic field signals over participants' scalps, in two observation conditions: observing a simple right-handed object manipulation performed either by a live actor, or on a pre-recorded video. In a third condition, participants performed the actions themselves. Järveläinen et al. (2001) found that the primary motor cortex showed corresponding activation during both observation and performance of actions. More importantly, they found that this activation was significantly reduced for the observation of video movements compared to live actions. Similar results have been observed in infants (Ruysschaert et al., 2013). Järveläinen et al. (2001) suggested that the difference between video and live feedback reflected the greater ecological validity of the latter and therefore greater participant interest in the 3D visual properties of the action. These results are particularly important considering recent findings suggesting that neural processes in interacting individuals may be “coupled” by contextual parameters (Schippers et al., 2010; Hasson et al., 2012; Yun et al., 2012). Hasson et al. (2012, p. 115) stated that “the coordination of behavior between the sender and receiver enables specific mechanisms for brain-to-brain coupling unavailable during interactions with the inanimate world.” If we are to measure social interaction, it seems best that we do indeed measure *interaction*, and not just *observation*. If we accept the commentary presented by Hasson et al. (2012), then social interaction is a “live” process, in which both parties are necessary to adequately represent the phenomenon.

Furthermore, most imitation research has used keypress or electromyographic measures from single effectors to measure imitation accuracy. Since muscle activity is only indirectly related to movement kinematics (Knudson, 2007), the above methods may not capture all the information encoded in movement. Perhaps surprisingly, few experiments have used motion tracking to study imitation, and most research has focused on the behavior of the imitator, rather than that of the actor or the interaction between the two. However, movement kinematics may help to inform the observer about an actor's intent (Becchio et al., 2008; Sartori et al., 2011; but see Naish et al., 2013), and the effect of movement observation on one's own actions can be so strong as to bias the action toward one more closely representing the observed action, even if imitation is not required (Hardwick and Edwards, 2012).

High-resolution motion tracking might allow greater insights into imitation, so the few studies using this methodology warrant attention. Wild et al. (2010) asked participants to observe videos of actors performing goal-directed and non-goal directed actions at fast and slow speeds and then to imitate the movements. A motion sensor was attached to the index finger and tracked in 3D. The participant's movement duration, peak velocity, and time to peak velocity, were then compared to the actor's kinematics. Campione and Gentilucci (2011) also used motion tracking to study whether the automatic imitation of reaching actions is effector dependent. They recorded peak velocity and peak acceleration of the wrist, thumb, and index finger as measures of the effects of action observation on movement planning. These studies extracted relatively few kinematic landmarks from relatively few body positions. A better approach might be to use the whole time-series from as many body parts as possible. The correlation between the time-series data of the actor's (the one performing the original actions) and the imitator's movements must necessarily provide a valid measure of imitation effectiveness and therefore a more ecologically valid observation of the imitation process. This was taken into account when designing our experiment.

Also worth consideration is the “correspondence problem” (for a detailed commentary see Brass and Heyes, 2005). It is still unclear how the brain is able to transform the visual parameters of an observed action into a motor output that can match it. This has been put forward as one potential role of the MNS and there is much discussion regarding whether or not it is the intended goal of an action that is imitated, or the entire movement profile itself. In our experiment, the choice was to focus on goal-directed, transitive (object-directed) imitation for two reasons. Firstly, because it allowed us to make use of a more naturalistic, variable task (as explained below), that did not rely on a number of pre-designated intransitive gestures. Secondly, this study was an attempt to explore the effects seen in experiments making use of object-directed imitation (e.g., Wild et al., 2010; Campione and Gentilucci, 2011; Braadbaart et al., 2012). This sort of imitation closely links to the learning of new motor skills, which occur throughout life, such as learning a new sport. Motion-tracking provides a reliable measure of this sort of imitation, since it can be used to test both object-directed task accuracy (the goal) and the kinematics as a whole.

The aim of the experiment reported here was twofold—first to understand what may be lost in typical transitive imitation paradigms using video feedback, and second to develop the use of motion tracking as a measure for examining imitation in pairs of people. By using face-to-face imitation we hoped to more closely measure imitation as it occurs relatively naturally. As such we developed an imitation game that allowed us to test participants on an object-directed task they are unlikely to have performed before. We recorded position data from 14 motion trackers distributed across the upper body and arms of six pairs of two participants, enabling us to greatly increase the number of tracked body locations compared to previous research. We then compared imitation accuracy in face-to-face feedback, and through a live video projection which prevented the imitator directly observing the actor. We hypothesized that video feedback would result in less accurate imitation than face-to-face feedback, and more complex imitation tasks would result in less accurate imitation than simple tasks. We developed analytic approaches to examine aspects of variable, dynamic time-series to look for correlations and their associated lags with regards to the movement and position of objects in the imitation task.

## Materials and methods

### Participants

Twelve right-handed participants (mean ± SE age = 29.4 ± 7.1 years, 2 male) were recruited from the University of Reading and the surrounding area. The experimental procedures were approved by the local ethics committee (refs: 2013_171_NH; UREC 11/11); participants gave written, informed consent; and the experiments were conducted in accordance with the Declaration of Helsinki. Each experiment required two participants, who took turns to perform as both actor and imitator.

### Apparatus and stimuli

The position of participants' heads, right arms and right hands were recorded continuously using a wired Polhemus Liberty (Polhemus Inc., Colchester, VT, USA) 240 Hz, 14 channel (7 per participant) motion tracking system with 6 degrees of freedom (x, y, z, azimuth, elevation, and roll). Trackers were attached to the shoulder (acromial end of clavicle), elbow (olecranon), wrist (pisiform), thumb (tip), index finger (tip), little finger (tip), and central forehead. Tracking points were attached using adhesive medical tape or Velcro™. The experiment was controlled and data were acquired using custom software written in MATLAB 2014b (Mathworks, Inc.) and using the ProkLiberty interface (https://code.google.com/p/prok-liberty/). We used LabMan and the HandLabToolbox to document and control experiments and analyze data. The associated repositories are freely available at https://github.com/TheHandLaboratory, whilst raw data are available from the Hand Laboratory's website (http://neurobiography.info) and/or on request.

**Figure 1:**
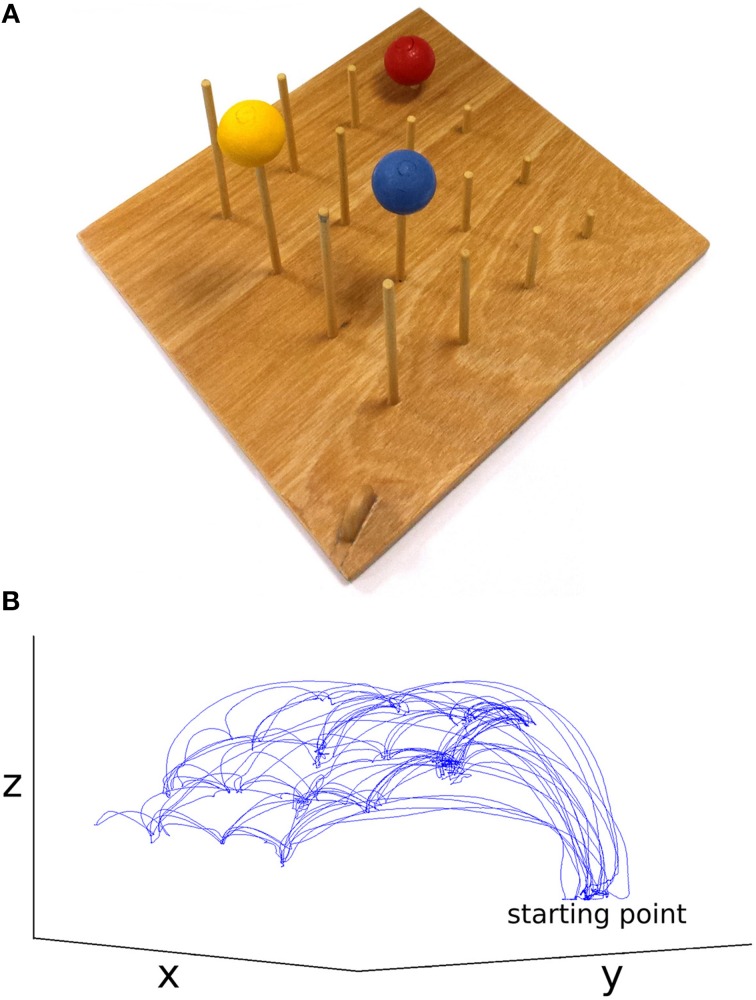
**(A)** Imitation game apparatus, starting point center bottom, **(B)** example dataset, movement of the thumb in x, y, and z for the face-to-face & simple condition (not to scale).

**Figure 2:**
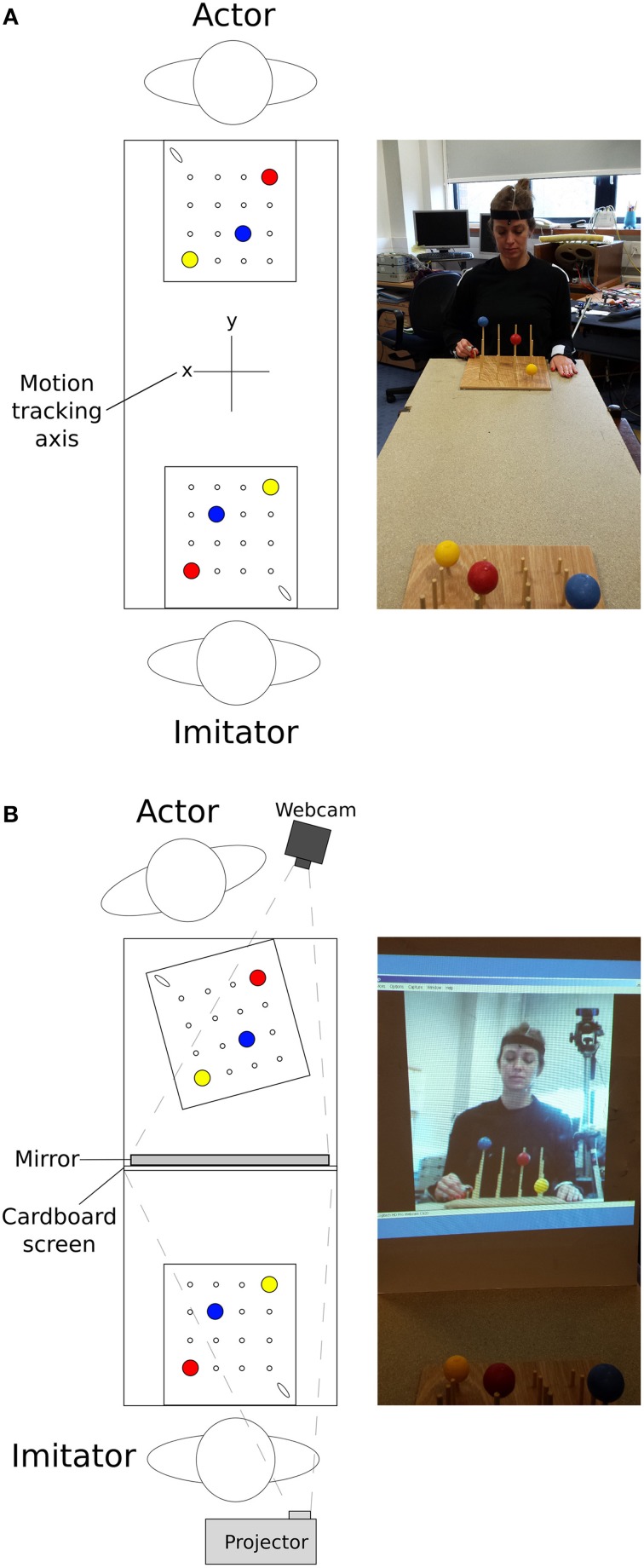
**Top-down view of the experimental set-up, along with an image of the approximate actor point of view for (A) face-to-face conditions and (B) video conditions; N.B. motion tracking axis in (B) is the same as in (A)**.

The stimuli used were two identical custom-designed wooden imitation games consisting of a 300 × 330 × 10 mm board with 4 × 4 vertical rods (diameter = 5 mm, 60 mm inter-rod spacing, Figure 1). The height of the 4 rods from front to back was 30, 70, 110, and 150 mm. On top of three of the rods were three colored (red, blue, yellow) solid cotton balls (diameter = 40 mm), with a 10 mm hole drilled into the center to allow rod placement. A curved wooden starting point of 30 × 8 × 25 mm was situated on the lower right corner near the tallest pegs. These boards were placed facing each other at opposite ends of a table approximately 1370 mm in length, at a distance of 710 mm apart (Figure 2). In all conditions the imitation game boards were attached securely to the table using Blu-Tak®. The Polhemus motion tracking transmitter was placed underneath the center of the table (not pictured in Figure 2).

Video conditions used a high definition webcam (Logitech International S.A., Switzerland) with a recording resolution of 1080p (resolution of 1920 × 1080, before zooming) and frame rate of approximately 30 FPS, to provide a live recording of the actor. A mirror was placed in front of the actor, angled at 70° to be visible by the camera which was positioned overlooking the actor's shoulder (Figure 2). The angled mirror was used to recreate a flat plane view of the actor in the video feed once the over-shoulder viewpoint was taken into account. A large white cardboard projection screen (840 × 590 mm) prevented the imitator viewing the actor. The webcam recorded the actor's movements from the mirrored image. This was then projected onto the cardboard screen (image size = 430 × 580 mm) for the imitator. The image was zoomed to the level that approximately represented the imitator's view of the actor in the face-to-face condition.

### Design

A repeated measures design was used, with two independent variables, each with two levels: task difficulty condition (simple, complex) and feedback condition (face-to-face, video). The task difficulty condition was used in order to test whether any effects of feedback condition depended on the complexity of the imitated actions—it was of interest to test whether more complex tasks would be more greatly affected by video feedback. The simple and complex conditions were tested once for each of the video or face-to-face conditions. Each participant played the role of both actor and imitator, meaning that each individual took part in a total of 2 sessions (80 trials)—one as an actor and one as an imitator, to account for two repetitions of the crossed condition design. Whilst using a single individual as the actor may have reduced variability between participants, we wanted to maintain a more naturalistic task with naïve participants, rather than a potentially biased confederate. Each crossed condition lasted 250 s and consisted of ten 20 s trials with 5 s rest gaps between. The dependent variables were the 6 degrees of freedom across 14 motion tracking points.

### Procedure

In each testing period, the two participants were assigned to either the role of actor or imitator, which were then reversed once 1 session (4 crossed conditions) was complete. Each testing session included a face-to-face and video feedback condition, and the order in which they occurred was randomized and counterbalanced (i.e., an imitator would observe and imitate in both the video and face-to-face conditions before swapping roles and becoming the actor). Both participants played both roles in order to maximize the data collected and ensure a balanced design. The simple difficulty conditions were always performed first in each feedback condition. This was done in place of a practice trial, in order to cut down testing time and maintain participant motivation and accuracy. Since we predicted that the simple task would be more accurately imitated anyway, we did not believe that this confound would be heavily altered by practice effects. The simple condition ensured that in each of the feedback conditions, the actor and imitator were quickly introduced to the constraints and demands of the task. Note that the main variable studied here is the feedback condition—face-to-face vs. video—the order of which was fully counterbalanced. A live video feed was used in the video feedback condition primarily to cut down on experimentation time, but also to reduce the variability between the feedback conditions to just the effects of video feedback.

In the face-to-face condition, participants sat opposite each other at either end of the table. The imitation boards were placed on the ends of the table in front of each participant who sat approximately 150 mm away. Both participants started with their right index finger and thumb gripping the starting point at the near right hand side of the board. The three balls were randomly distributed across the pegs on the actor's game board at the start of each condition, and the imitator's game board was matched to this. The actor was requested to move balls across the board in two different conditions, whilst the imitator copied the actions in an anatomical fashion (i.e., both participants used their right hand, and a move of the ball to the right by the actor corresponded to a move of the ball to the anatomical right for the imitator), as accurately as possible. Anatomical imitation was used to maintain a more naturalistic imitation task. This is akin to what may happen when one right-handed individual teaches another right-handed individual to perform a motor task, rather than in instances of spontaneous imitation where a mirrored response is more likely to be used by the observer (Pierpaoli et al., 2014).

In the simple condition, the actor freely moved a single ball along 10 consecutive and adjacent pegs moving left or right, or up or down, but not diagonally, touching each peg with the ball before placing it on the peg reached once 10 moves were complete. They then returned to the starting point, gripping it with thumb and index finger. The complex condition also required 10 moves across consecutive pegs, but in this case participants were required to use each of the three balls, in any order as long as a total of 10 moves were made. In each of the crossed conditions, the actor was permitted to move the balls freely within the given parameters of the task, and did not have to perform the same movement sequence across different conditions. Both the actor and the imitator were informed of the constraints of the actor's task. A beep played through the computer's speakers signaled the actor and imitator to begin and finish at the start and end of each 20 s trial. Participants were requested to make the most of the total 20 s, timing their 10 moves accordingly. Participants always moved back to the start point once their moves were complete. Example data are shown in Figure 1. Imitators were requested to copy the actor's movements as accurately as possible. They were asked to begin imitating the actor as soon as the actor started moving. No instructions were given to either participant regarding eye gaze.

The tasks in the video feedback condition were identical, except that the imitator observed the actor through a live video projection, and any natural vision of the actor was obscured by the cardboard screen (Figure 2). For the actor, the angle of the imitation game was shifted by 13° anticlockwise and the apex of the mirror was placed 570 mm from the edge of the table, with the reflective side facing the actor. The actor was then seated facing the game board at the same distance and orientation as in the video condition (i.e., directly facing the board, sat approximately 150 mm away). These changes allowed the webcam (angled appropriately) to record the actions of the actor, passing the video on to an image projected on to the card screen mounted on the back of the mirror, 640 mm away from the imitator. The imitator could perform the required actions without direct observation of the actor.

At the start of each video or face-to-face condition, a brief calibration test was run. This required the actor to trace the outside of the imitation game board with their thumb and index finger, following a tone. The imitator was requested to copy this action. The calibration enabled the experimenter to ensure that all trackers were recording correctly and that there were no obvious distortions in the data prior to data collection.

### Data pre-processing

Five pre-processing steps were performed in order to clean the data. First, single time-point spikes (>3 SD from the mean) in each variable's double-differentiated time-series (i.e., acceleration) were deemed electromagnetic artifacts and removed by interpolation across two adjacent samples either side. Second, the position data were filtered using a bidirectional low-pass 4th order Butterworth filter (cutoff frequency 15 Hz). Third, the position data for the actor in the video condition were rotated by 13° clockwise in the x (*x* = *x*(cos 13) − *y*(sin 13)) and y (*y* = *y*(cos 13) + *x*(sin 13)) axes in order to correct for the angled game board.

Fourth, the time-series for the imitator data in the video condition was shifted backwards by 111 ms to account for the latency between the recording and presentation of video stimuli, ensuring that any effects of the video condition were due to the condition itself rather than the delay in stimulus presentation. Latency was calculated by measuring the time difference on an independent PC using Chart 5 software to detect a flash of light presented to two light detecting diodes—one located at the webcam aperture, the second located on the cardboard screen used to project video stimuli. Diodes were connected via a custom interface to an AD Instruments data acquisition unit sampling at 2 kHz. Video latency (the time between light detection in each of the two diodes) was measured over 25 discrete tests (whilst the data collection script was running in the background to simulate the experimental condition), resulting in a mean ± SD latency of 111 ± 25 ms.

Finally, since data collection was continuous during the entire length of the condition (including rests) and actors often finished their 10 movements before the end of the (20 s) trial time, the lengths of each trial were calculated independent of the total trial time. This was done by defining correct trials (i.e., ignoring false starts) as >100 mm movement of the index finger away from the start point for any period >5 s. This ensured that false starts were excluded from the analysis, and trial onsets were timed to the actors' movements. These variable trial times were also applied to each actor's associated imitator's data, since imitators were requested to begin movement at the same time as the actor.

### Exploratory data analysis

Prior to full data processing, an exploratory analysis of one half of the data (3 pairs of participants) was performed. This was deemed necessary due to the novel methods developed in this experiment, as well as the potential for false positives with such a large dataset and so many dependent variables. We hoped that it would reveal any consistent effects across degrees of freedom, and direct our choice of final analysis parameters based on this. Each crossed condition (task difficulty × feedback condition) yielded 42 dependent variables for each participant (84 in total): 7 motion trackers × 6 degrees of freedom (x, y, z, azimuth, elevation, roll).

**Figure 3:**
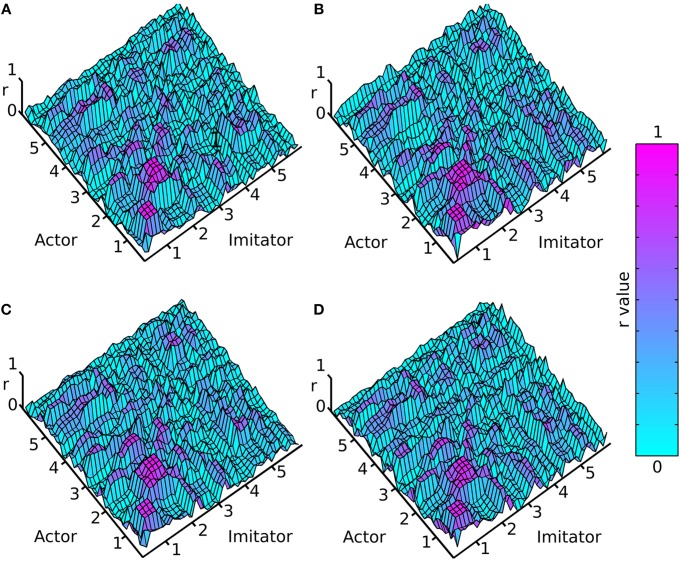
**Mean absolute maximum** ***r***-**value (colorbar = 0:1 r), (A) face-to-face & simple, (B) face-to-face & complex, (C) video & simple, (D) video & complex; x and y axes represent actor and imitator trackers within their degrees of freedom (head, shoulder, elbow, wrist, thumb, index finger, little finger, in x, y, z, azimuth, elevation, and roll): 1 = x, 2 = y, 3 = z, 4 = azimuth, 5 = elevation, 6 = roll**.

**Figure 4:**
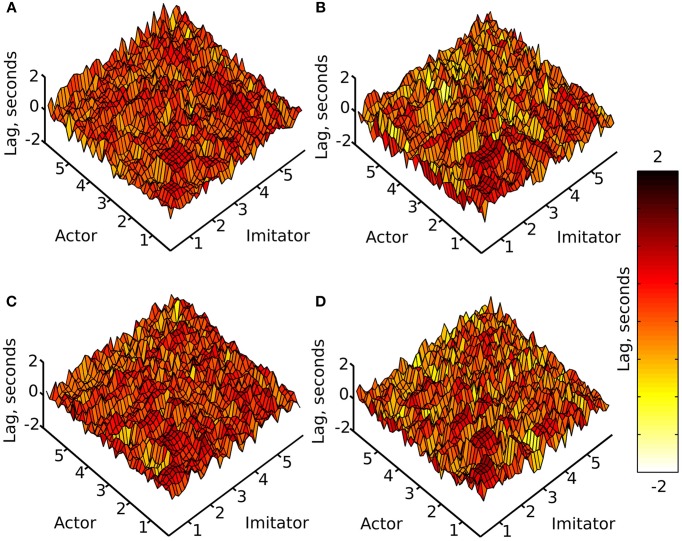
**Mean lag at absolute maximum** ***r***-**value (colorbar = −2:+2 s), (A) face-to-face & simple, (B) face-to-face & complex, (C) video & simple, (D) video & complex; x and y axes represent actor and imitator trackers within their degrees of freedom (head, shoulder, elbow, wrist, thumb, index finger, little finger, in x, y, z, azimuth, elevation, and roll): 1 = x, 2 = y, 3 = z, 4 = azimuth, 5 = elevation, 6 = roll**.

A cross-correlation was performed on each of the crossed conditions over each of the 10 (variable length—see data pre-processing) trials. This was done by shifting the imitator's data relative to the actor's sample by sample over lags of −5 to +5 s, and correlating the two time-series for each lag (−1200 to +1200 samples). For each of these 10 trials, an absolute maximum *r*-value between each actor dependent variable and each imitator dependent variable was generated, along with the lag associated with that maximal *r*-value (as a measure of the best-fitting overall lag between actor and imitator). The lag at maximum r represents the difference (in time) between the actor and imitator datasets at the point at which the maximum *r*-value was found. These results were averaged across the 10 action trials per participant and then across the 6 participants to generate the surface plots in Figures 3, 4.

The surface plots suggested that absolute maximum *r*-value and the lag associated with it varied widely across dependent variables. The most consistently highly correlated values were the corresponding trackers in their corresponding degrees of freedom. This was emphasized by the highly correlated diagonal contours in the surface map of *r*-values in Figure 3 (particularly in x, y, and z). The greater density of pink coloring in the face-to-face condition *r*-value plots seemed to suggest that it may be better correlated than the video condition; however it was hard to gauge any large differences between correlations in the difficulty conditions. The surface plots in Figure 4 suggested that the lag associated with the maximal *r*-value was, surprisingly, lower in the complex vs. the simple conditions. It also appeared that the video conditions may have had slightly lower lags than the face-to-face conditions, though this was less clear.

### Final analysis parameters

Based on the exploratory analysis it was decided that an analysis of the entire dataset (12 participants) would benefit from parameters that capture the greatest movement information in the fewest dependent variables. As such, we decided to focus on three elements of the task: joint angles in the arm, grip aperture, and grip position, each of which were calculated for actor and imitator. This analysis was performed on all 12 participants' data. Joint angles of the arm were selected because the angles of all the joints in any given effector across time provide a general representation of the whole movement. Thus, by examining the joint angles between the trunk, shoulder, elbow and wrist, it was possible to develop a reasonably accurate measure of the entire arm movement. This would enable us to compare kinematic, rather than goal outcome accuracy of the imitator.

**Figure 5:**
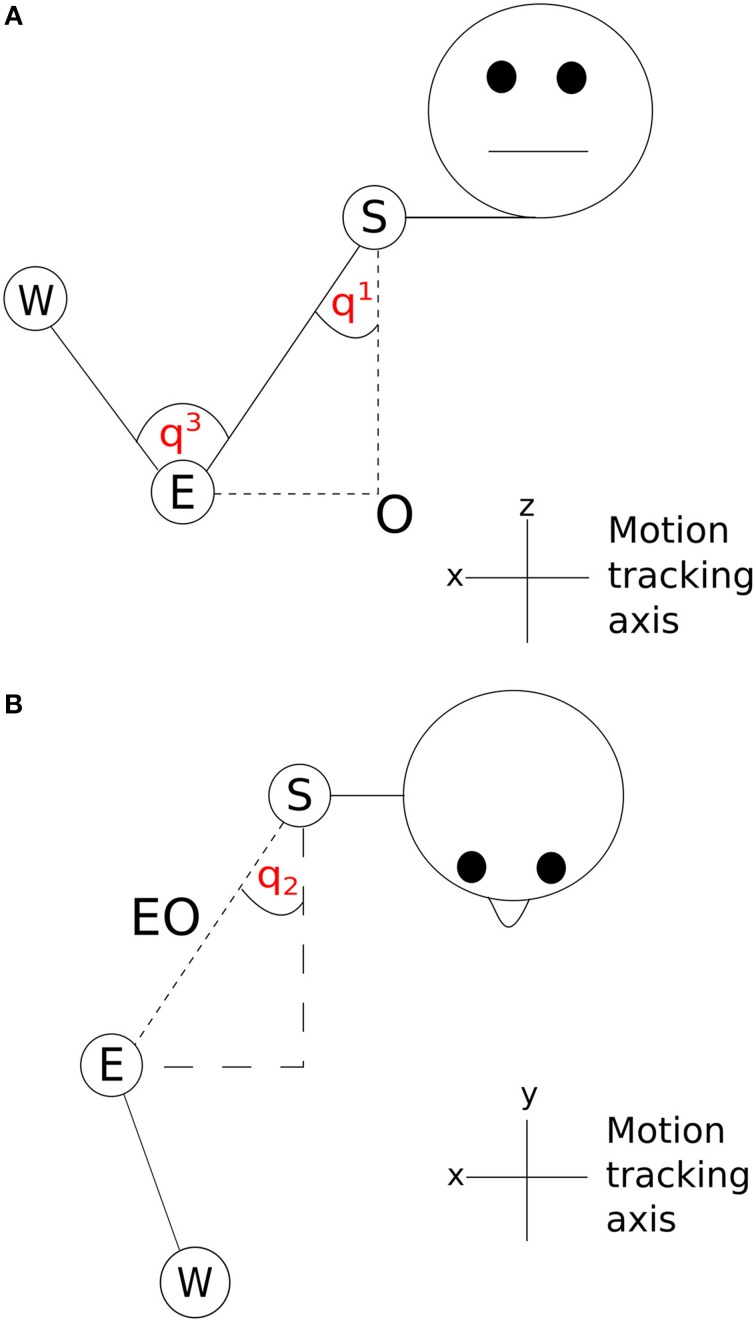
**Visualization of joint angles (A) q_1_ and q_3_ and (B) q_2_; S, shoulder; E, elbow; W, wrist; O, origin**.

The two angles between the shoulder and the body in the x and y dimensions (q_1_ and q_2_) are shown in Figure 5. A vector **SO** starting at the shoulder, S and ending at the origin, O was determined by subtracting the z dimension position value of the elbow from the z dimension position value of the shoulder. By using this vector along with the elbow-shoulder vector **ES**, a right angle triangle was formed. Angle q_1_ was calculated as the angle between vectors **ES** and **SO** $\left(q_{1}=\cos^{-1}\left(\frac{z_{shoulder}-z_{elbow}}{ES}\right)\right)$. A projection of the vector **EO** between the elbow and origin was created in the x and y dimensions. In the x and y dimension a second right angle triangle was created using the vector **EO** and a second vector calculated by subtracting the y dimension position of the elbow from the y dimension position of the shoulder. q_2_ was calculated as the angle between **EO** and this second vector $\left(q_{2}=\cos^{-1}\left(\frac{y_{shoulder}-y_{elbow}}{EO}\right)\right)$. The inner elbow angle q_3_ (Figure 5) was calculated through the cosine rule, taking the elbow-to-wrist **EW** and elbow-to-shoulder **ES** as two intersecting vectors $\left(q_{3}=\cos^{-1}\left(\frac{SW^{2}-ES^{2}-EW^{2}}{2\left(ES\times EW\right)}\right)\right)$. Using joint angles in this way reduced the number of position parameters to examine from nine (3 tracking points × 3 axes) to three (3 angles, q_1_–q_3_).

We also used the grip aperture of the index finger and thumb. Grip aperture is a commonly recorded parameter in kinematics (Castiello and Ansuini, 2009), and provides a measure of the primary movement required for this task. The grip aperture variable was created by calculating the 3D distance between the index finger and the thumb. Finally, the grip position was recorded. This was done by taking the mean location of the index finger and thumb in x, y, and z. We hoped that this would provide a general measure of task imitation accuracy, rather than movement imitation accuracy, since some authors have claimed that it is the goals of an action that are imitated, rather than the means (Wohlschläger et al., 2003).

These three new DVs were cross-correlated in an identical manner to the exploratory analysis, resulting in absolute maximum *r*-values and their associated lags for each of the trials across each of the crossed conditions. For participants 11 and 12, the final trial of the complex face-to-face condition was excluded due to the actor's (participant 11) failure to return their hand to the starting point. The means of the *r*-values and lags across trials was calculated to provide 7 DVs (q_1_, q_2_, q_3_, grip aperture, grip position in x, y, and z) for each participant across the two experimental conditions. For each of these new DVs mean *r*-values between participants across the 10 trials per crossed condition were converted to *Z*-values using the Fisher transformation $\left(Z=\frac{1}{2}\text{ln }\left(\frac{1+\text{r}}{1-\text{r}}\right)\right)$, where ln is the natural logarithm of a number. This allowed parametric statistics to be used on the *r*-values.

## Results

**Figure 6:**
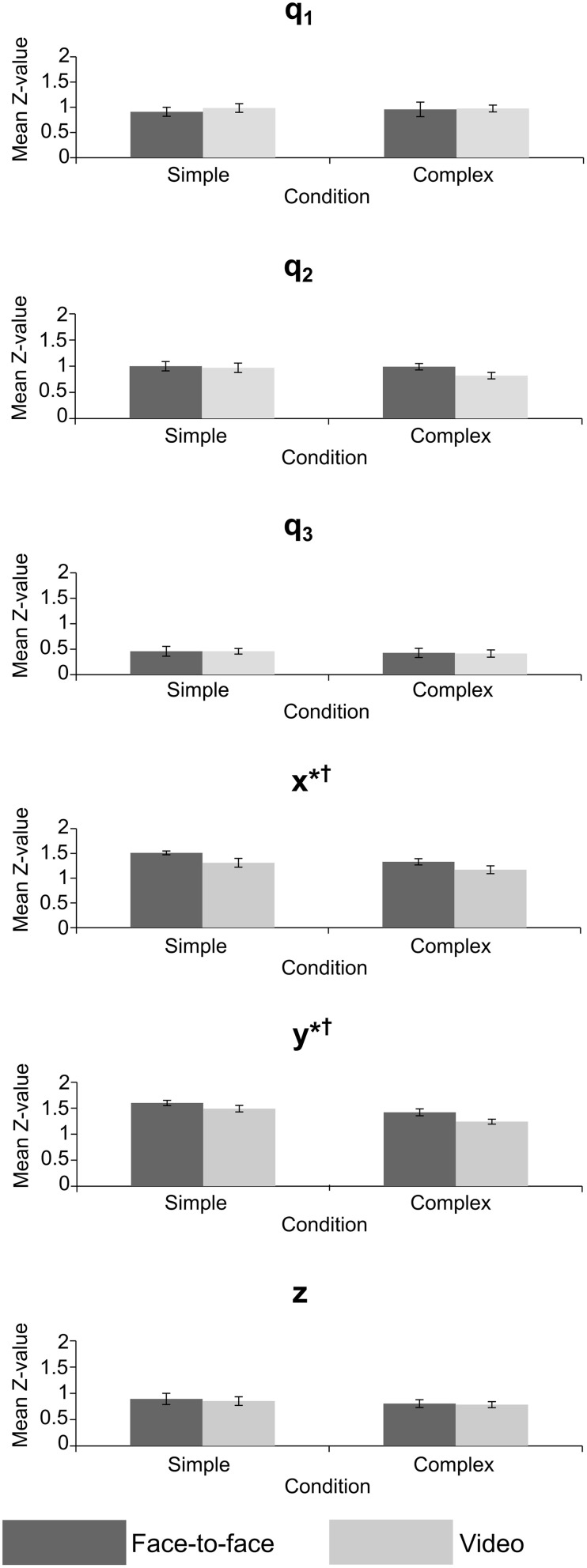
**Mean** ***Z***-value for crossed conditions in the joint angle and grip position variables, error bars = standard error; ^*^significant effect of feedback, ^†^significant effect of task difficulty. *p* < 0.05 for all significant effects—see Table 1 for exact values.

**Figure 7:**
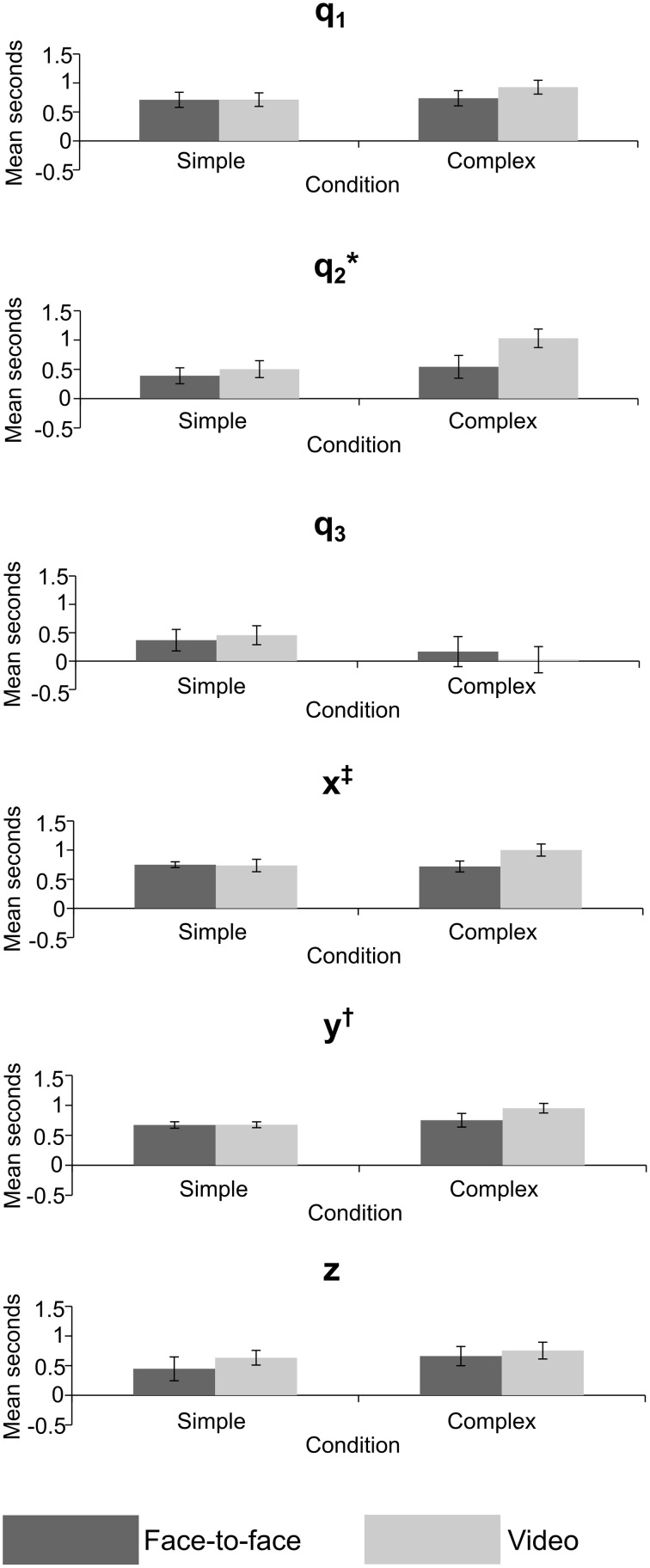
Mean lag in seconds for crossed conditions in the joint angle and grip position variables, error bars = standard error; ^*^significant effect of feedback, ^†^significant effect of task difficulty, ^‡^significant interaction. *p* < 0.05 for all significant effects—see Table 2 for exact values.

Repeated measures MANOVAs were run on the *Z*-values and lags at absolute maximum *r*-value, for joint angles (q_1_–q_3_) and grip position (x, y, z). A Two-Way repeated measures ANOVA was run on the *Z*-values and lags at absolute maximum *r*-value for the grip aperture values. The MANOVAs and ANOVA compared the mean *Z*-value and mean lag of the 10 trials between the feedback and difficulty conditions across all 12 experiments (24 sessions). The results of the MANOVAs are given in Tables 1, 2, and mean values are shown in Figures 6, 7.

**Table 1:** **MANOVA and ANOVA main effects and interactions for mean *Z*-value**.

		**Mean(±SE) *Z*-value**				**Main effect**						Feedback^*^difficulty interaction		
		**Feedback**		**Task difficulty**										
		**Face-to-face**	**Video**	**Simple**	**Complex**	**Feedback**			**Difficulty**			F^*^	***p***	**Partial η^2^**
						F^*^	***p***	**Partial η^2^**	F^*^	***p***	**Partial η^2^**			
Joint angle	Multivariate	0.791	0.770	0.797	0.764	1.27	0.344	0.297	1.29	0.337	0.300	0.916	0.471	0.234
		(0.062)	(0.045)	(0.052)	(0.055)									
	q_1_	0.934	0.980	0.947	0.967	0.327	0.579	0.029	0.105	0.751	0.009	0.245	0.631	0.022
		(0.111)	(0.067)	(0.083)	(0.092)									
	q_2_	0.995	0.894	0.984	0.905	2.16	0.170	0.164	1.39	0.264	0.112	1.90	0.195	0.147
		(0.067)	(0.061)	(0.074)	(0.051)									
	q_3_	0.443	0.436	0.458	0.421	0.008	0.930	0.001	0.866	0.372	0.073	0.014	0.909	0.001
		(0.085)	(0.058)	(0.061)	(0.065)									
Grip position	Multivariate	1.26	1.14	1.28	1.13	3.32	0.071	0.529	7.32	**0.009**	0.709	0.145	0.930	0.046
		(0.041)	(0.029)	(0.045)	(0.035)									
	x	1.42	1.24	1.41	1.25	9.41	**0.011**	0.461	6.27	**0.029**	0.363	0.126	0.729	0.011
		(0.047)	(0.061)	(0.057)	(0.054)									
	y	1.51	1.37	1.55	1.33	6.77	**0.025**	0.381	13.8	**0.003**	0.557	0.216	0.651	0.019
		(0.043)	(0.029)	(0.040)	(0.035)									
	z	0.850	0.819	0.874	0.795	0.198	0.665	0.018	0.842	0.379	0.071	0.025	0.887	0.002
		(0.079)	(0.038)	(0.076)	(0.057)									
Grip aperture		0.186	0.142	0.180	0.149	0.310	0.589	0.027	0.389	0.545	0.034	0.165	0.693	0.015
		(0.063)	(0.072)	(0.043)	(0.074)									

* *Degrees of freedom for multivariate analyses are (3, 9), for univariate (1, 11); significant p-values (<0.05) are in bold*.

**Table 2:** **MANOVA and ANOVA main effects and interactions for mean lag**.

		**Mean lag(±SE) in seconds**				**Main effect**						Feedback^*^difficulty interaction		
		**Feedback**		**Task difficulty**										
		**Face-to-face**	**Video**	**Simple**	**Complex**	**Feedback**			**Difficulty**			F^*^	***p***	**Partial η^2^**
						F^*^	***p***	**Partial η^2^**	F^*^	***p***	**Partial η^2^**			
Joint angle	Multivariate	0.485 (0.096)	0.609 (0.082)	0.523 (0.085)	0.572 (0.108)	1.87	0.206	0.383	2.67	0.110	0.471	0.508	0.687	0.145
	q_1_	0.722 (0.106)	0.820 (0.085)	0.711 (0.080)	0.831 (0.096)	0.590	0.458	0.051	1.43	0.257	0.115	0.578	0.463	0.050
	q_2_	0.466 (0.120)	0.769 (0.111)	0.447 (0.112)	0.789 (0.138)	5.57	**0.038**	0.336	4.48	0.058	0.289	1.56	0.237	0.124
	q_3_	0.267 (0.198)	0.240 (0.158)	0.412 (0.141)	0.095 (0.203)	0.016	0.902	0.001	2.36	0.152	0.177	0.823	0.384	0.070
Grip position	Multivariate	0.666 (0.071)	0.792 (0.073)	0.652 (0.051)	0.807 (0.069)	0.673	0.590	0.183	3.95	**0.047**	0.586	2.24	0.153	0.427
	x	0.734 (0.062)	0.869 (0.096)	0.742 (0.060)	0.861 (0.076)	1.69	0.22	0.133	3.70	0.081	0.252	5.93	**0.033**	0.350
	y	0.712 (0.077)	0.814 (0.056)	0.674 (0.040)	0.852 (0.078)	1.70	0.219	0.134	10.7	**0.007**	0.494	2.80	0.122	0.203
	z	0.553 (0.138)	0.694 (0.113)	0.540 (0.098)	0.707 (0.114)	0.51	0.49	0.044	1.38	0.265	0.112	0.122	0.733	0.011
Grip aperture		0.043 (0.201)	0.054 (0.211)	0.275 (0.171)	−0.178 (0.262)	0.003	0.955	<0.001	3.55	0.086	0.244	0.163	0.694	0.015

* *Degrees of freedom for multivariate analyses are (3, 9), for univariate (1, 11); significant p-values (<0.05) are in bold*.

The MANOVA on *Z*-values (Table 1), measuring the strength of correlation between actor and imitator, revealed 5 significant effects. Both the x [*F*_(1, 11)_ = 9.41, *p* = 0.011, partial η^2^ = 0.461] and y [*F*_(1, 11)_ = 6.77, *p* = 0.025, partial η^2^ = 0.381] grip positions showed a significant effect of feedback, with the face-to-face condition more highly correlated than the video condition (mean ± SE difference in *Z*-values = 0.179 ± 0.058 for x, and 0.145 ± 0.056 for y data), providing some support in favor of our hypothesis. The mean *Z*-values for x were equivalent to *r*-values of 0.889 for face-to-face feedback and 0.845 for video feedback. For y the equivalent *r*-values were 0.907 for face-to-face feedback and 0.878 for video feedback. Both the x [*F*_(1, 11)_ = 6.27, *p* = 0.029, partial η^2^ = 0.363] and y [*F*_(1, 11)_ = 13.8, *p* = 0.003, partial η^2^ = 0.557] grip positions showed significant effects of task difficulty, with the simple condition more highly correlated than the complex (mean ± SE difference in *Z*-values = 0.158 ± 0.063 for x, and 0.215 ± 0.058 for y). The mean *Z*-values for x were equivalent to *r*-values of 0.887 for simple task difficulty and 0.848 for complex task difficulty. For y the equivalent *r*-values were 0.913 for simple task difficulty and 0.870 for complex task difficulty. These two significant univariate effects also resulted in a significant multivariate effect in multivariate grip position for task difficulty, *F*_(3, 9)_ = 7.32, *p* = 0.009, partial η^2^ = 0.709. The mean *Z*-values for this multivariate variable were equivalent to *r*-values of 0.856 for simple task difficulty and 0.811 for complex task difficulty.

The lag MANOVA (Table 2) revealed 4 significant effects. There was a significant effect of feedback in joint angle q_2_, *F*_(1, 11)_ = 5.57, *p* = 0.038, partial η^2^ = 0.336, with the video condition showing a longer delay than the face-to-face (mean ± SE difference = 0.302 ± 0.128 s). The multivariate grip position was significant for task difficulty, *F*_(3, 9)_ = 3.95, *p* = 0.047, partial η^2^ = 0.586, with the complex condition significantly more delayed than the simple (mean ± SE difference = 0.155 ± 0.053 s). The y grip position also showed a significant effect of task difficulty, *F*_(1, 11)_ = 10.7, *p* = 0.007, partial η^2^ = 0.494, with the complex condition significantly slower than the simple (mean ± SE difference = 0.178 ± 0.054 s). Finally, there was a significant interaction between task difficulty and feedback in the x grip position, *F*_(1, 11)_ = 5.93, *p* = 0.033, partial η^2^ = 0.350, where simple conditions showed longer imitation lags than complex when observed face-to-face (mean ± SE difference = 0.031 ± 0.087 s), but imitation in the complex conditions was later than the simple when observed via video (mean ± SE difference = 0.268 ± 0.087 s).

## Discussion

We examined the effects of face-to-face vs. video feedback on imitation in a transitive imitation task, hypothesizing that video feedback would result in less accurate imitation and that a simpler task would result in more accurate imitation than a complex one. After running an exploratory analysis, we chose to perform a more focused statistical analysis on grip position, joint angles in the arm, and grip aperture.

In the correlation (*Z*-value) analysis, only the grip position variables revealed significant effects of feedback and task complexity. Grip position can be taken as a general measure of accuracy in our imitation task, since it measures the position of the object effectors (index finger and thumb) from the starting point, across the movement of the balls, and then the return of the hand to the starting point. The significant differences suggested that video feedback reduced the accuracy of transitive imitated actions for left-right (x) and forward-back (y) dimensions of motion, but not for up-down (z). This supports our hypothesis that video feedback would be less highly correlated than face-to-face observation. Imitators were worse at completing the imitation task when required to view the actor through a live video feed. The source of this effect is most likely the difference in visual information provided by the video and face-to-face feedback conditions, but it is also possible that increased motivation driven by the ecological validity of the face-to-face condition is responsible (Järveläinen et al., 2001). However, the continued presence of the actor in the room during both feedback conditions suggests either that this explanation is lacking, or that such an effect may be strong enough to compensate for the imitator's knowledge about the actor's location. These are important findings when considering previous imitation research that has used video stimuli, particularly for studies using object-directed actions. At the very least these studies have not accounted for the effect of visual feedback and may be lacking in ecological validity. It is likely that imitation was altered in these studies, with accuracy being reduced by video feedback.

Comparing simple and difficult tasks, the forward-back and left-right dimensions of grip position also showed significant effects, with the simple task more highly correlated than the complex one, suggesting our manipulation of task difficulty was effective. The lack of significant interactions between feedback and difficulty in the correlation analyses suggests that the effects of face-to-face vs. video feedback were not affected by task complexity.

Despite the significant results in the grip position analysis, grip aperture and joint angles showed no such effects. This may be the result of imitators copying the motion of the ball (the goal), but failing to imitate the broader motion of the actor's arm. This is likely due to our use of a transitive task, and may lend credence to claims that transitive imitation is primarily goal-directed, and that it is the object of the goal that is imitated, rather than the associated body movements (Wohlschläger et al., 2003; but see Leighton et al., 2010). However, a number of other factors may have influenced this outcome. It may also be due to our use of anatomical, rather than mirror imitation, or the fact that imitators had to shift their attention between the actor's game board and their own, thus limiting the resources available to imitate movements outside of the task constraints. In addition, grip aperture showed no effects of feedback or difficulty. This may be because the proportion of time that grip aperture was changing was too low to detect significant effects. When both actor and imitator were holding a ball, there was no longer a time-varying correlation between their (constant) grip apertures.

What remains to be explained from the correlation analysis is why the grip position in the forward-back and left-right directions were significant, whilst up-down was not. One explanation is that up-down movements were not influenced by the effects of the video condition. Certainly up-down movements of the balls were clearer to observe in the video condition than forward-back. Movements forward-back were hard to distinguish in the video condition without depth information (i.e., pegs that were lined up in front of each other were less distinguishable compared to those going left to right). However, the up-down effects were in the same direction as other dimensions (Figure 6), suggesting that the effect was too weak to be detected. The absence of significant effects for joint angles and grip aperture may indicate that some aspects of object-directed imitation are not strongly affected by video feedback. Eye-tracking could have been useful in this respect. Measurement of imitator eye movements could have shown whether they were concentrating on the actor's movements in general, rather than the end point of the ball (the goal).

The results of the lag analysis were less consistent than the correlation analysis. The most interesting result was for joint angle q_2_—the rotation of the upper arm about the shoulder—where imitation was significantly later in the video than face-to-face condition. This may be related to the reach-to-grasp action, and the difference in lag between face-to-face and video conditions may reflect a delayed approach toward the balls by the imitator. This could again be related to the ecological validity or motivation in the video condition. The significant multivariate effect for grip position suggests that overall, imitators acted later to accurately imitate the ball movements in the complex condition. The same effect was also shown in univariate analysis for the forward-back movements, meaning that they were imitated more slowly in complex tasks, potentially reflecting a greater use of this dimension in complex tasks (i.e., for the actor to move their hand to other balls). Movement of grip position left-right showed a significant interaction. Whilst the effect of the video condition was in the predicted direction, the difference in the face-to-face condition for left-right movement may be due to a better level of prediction by the imitators for complex rather than simple conditions in this direction, though it is unclear why this would be the case.

The differences between face-to-face and video observation may partly be due to the ±25 ms SD in the video projection latency. This temporal jitter surprised us, and was not controlled for in our experiment or analysis. This variable is also likely not controlled in previous research using pre-recorded video stimuli, such that researchers cannot be sure of a constant level of visual quality in their stimuli. Varying visual quality at any one time in a video could alter participant responses in a way that is not consistent with the variable being measured. We believe that researchers would benefit from providing this measure of standard deviation, or some other measure of temporal precision of video stimuli.

Some aspects of our experimental approach may have limited the reliability and validity of our results. Allowing actors to move in any way they chose, rather than in 10 consecutive movements, may have resulted in data more indicative of real life transitive motor activity. However, we felt it was important to maintain some element of control over the way in which participants moved for a number of reasons. By providing a relatively fixed way in which the actor was required to move, it ensured that their actions had a specific aim. As mentioned above, intention is potentially important in action observation (Becchio et al., 2012), and allowing the actor to move completely freely may have resulted in changes in their aims across conditions. Secondly, we believed that having a set aim across the trials better reflected imitation in real life tasks that have a definite goal and action profile (for example, serving in a game of tennis). This paradigm also ensured that trials could be compared to each other across participants and conditions with reasonable accuracy.

Additionally, a confound in the order of the difficulty conditions may have affected the results with regards to practice effects, but if practice effects were strong, the effects should be in the opposite direction to those found. Using the same participants as both actor and imitator may also have affected the results, with participants playing the role of actor first potentially displaying greater skill at the imitation task. However, an even number of participants ensured that condition order was counterbalanced. Two out of the 12 participants tested were male, and differences in gender may have in some way influenced the results, since there is evidence for differences in simulation strategies between males and females (Kessler and Wang, 2012).

Lastly, our treatment of joint angles, though novel in the research of imitation kinematics, was not entirely optimal. First and foremost, q_1_–q_3_ were not “true” joint angles in that they did not pass through the center of the joints. This was impossible to avoid with motion trackers on the surface of the skin, and has been commented on before by previous (non-imitative) research using joint angle kinematics (e.g., Murphy et al., 2006). We do not believe that this undermines the analysis, since the joint angle calculations can be seen as a best estimate, and are likely to closely resemble the true joint angle motion of the actor and the imitator. In addition to this, q_3_ did not take into account the rotation of the wrist. However, since we used joint angles as a general measure of arm movement, and not as a way to define the position of the hand, this was also of little concern to our analysis.

Future research may choose to focus on neural differences between face-to-face and video feedback in transitive imitation. This is especially timely considering it is 14 years since Järveläinen et al. (2001) found measurable differences in motor cortex activity between observation of motor actions in face-to-face and video stimuli. Changes in the activity of the motor cortex are likely accompanied by changes in regions including the inferior frontal gyrus, inferior parietal lobule, and posterior superior temporal sulcus (Molenberghs et al., 2009). Translating our design to neuroimaging or neurostimulation may further develop our understanding of the neural effects of video feedback. Another avenue for research could aim to discover where the difference between face-to-face and video feedback lies. Is it due to the lack of real two-person interaction, or rather due to visual differences between video and real life observation? The findings of Järveläinen et al. (2001) suggest that it could be the latter, but there is a growing consensus regarding the importance of two-person interactions in social psychological research (Schippers et al., 2010; Yun et al., 2012; Liu and Pelowski, 2014). In this experiment the difference could also be due to the reduced social context available to the actor. Perhaps a more reliable way of using pre-recorded video stimuli in the future would involve videoing an actor in an actual imitation task, rather than just performing actions of their own accord (though this could create new problems). As mentioned in the introduction, it is still unclear how an observer can constrain their own motor system in order to imitate an action (the correspondence problem). Our experiment suggests that this process may be influenced in some way by variables beyond simple motor observation, such as the visual quality of the observed movement or the extent to which it is likely to result in a real, two-person interaction. This is worth considering when testing different aspects of imitation. Social aspects of imitation may be more influenced by the lack of real face-to-face interaction, whilst motor aspects may be more influenced by the visual fidelity of video stimuli.

To conclude, it is evident that there are detrimental effects of video stimuli on the accuracy of imitation which may have been overlooked in previous research. This is evident in positional information regarding task-specific, object-directed movement. However, other aspects of transitive imitation (joint angles, grip aperture), may not be affected by the use of video stimuli. Future research should aim to develop new methods of examining imitation that are less reliant on video stimuli, and more closely adhere to the idea of imitation as a method of social communication. This would ensure the development of a more complete understanding of human imitation.

### Conflict of interest statement

The authors declare that the research was conducted in the absence of any commercial or financial relationships that could be construed as a potential conflict of interest.
